# GDF15 Plasma Level Is Inversely Associated With Level of Physical Activity and Correlates With Markers of Inflammation and Muscle Weakness

**DOI:** 10.3389/fimmu.2020.00915

**Published:** 2020-05-12

**Authors:** Maria Conte, Morena Martucci, Giovanni Mosconi, Antonio Chiariello, Maria Cappuccilli, Valentina Totti, Aurelia Santoro, Claudio Franceschi, Stefano Salvioli

**Affiliations:** ^1^Department of Experimental, Diagnostic and Specialty Medicine (DIMES), University of Bologna, Bologna, Italy; ^2^Nephrology and Dialysis, Morgagni-Pierantoni Hospital, AUSL Romagna, Forlì, Italy; ^3^Department of Biomedical and Neuromotor Sciences, University of Bologna, Bologna, Italy; ^4^Laboratory of Systems Medicine of Healthy Aging and Department of Applied Mathematics, Lobachevsky University, Nizhny Novgorod, Russia

**Keywords:** GDF15, physical activity, sedentarity, inflammation, skeletal muscle, healthy aging

## Abstract

Growth differentiation factor 15 (GDF15) is a stress molecule produced in response to mitochondrial, metabolic and inflammatory stress with a number of beneficial effects on metabolism. However, at the level of skeletal muscle it is still unclear whether GDF15 is beneficial or detrimental. The aim of the study was to analyse the levels of circulating GDF15 in people of different age, characterized by different level of physical activity and to seek for correlation with hematological parameters related to inflammation. The plasma concentration of GDF15 was determined in a total of 228 subjects in the age range from 18 to 83 years. These subjects were recruited and divided into three different groups based on the level of physical activity: inactive patients with lower limb mobility impairment, active subjects represented by amateur endurance cyclists, and healthy controls taken from the general population. Cyclists were sampled before and after a strenuous physical bout (long distance cycling race). The plasma levels of GDF15 increase with age and are inversely associated with active lifestyle. In particular, at any age, circulating GDF15 is significantly higher in inactive patients and significantly lower in active people, such as cyclists before the race, with respect to control subjects. However, the strenuous physical exercise causes in cyclists a dramatic increase of GDF15 plasma levels, that after the race are similar to that of patients. Moreover, GDF15 plasma levels significantly correlate with quadriceps torque in patients and with the number of total leukocytes, neutrophils and lymphocytes in both cyclists (before and after race) and patients. Taken together, our data indicate that GDF15 is associated with decreased muscle performance and increased inflammation.

## Introduction

Growth differentiation factor 15 (GDF15), also known as macrophage inhibitory cytokine 1 (MIC-1), is a stress responsive member of the transforming growth factor-β (TGF-β) cytokine superfamily, discovered in 1997 (1). GDF15 modulates appetite and energy metabolism possibly by regulating mitochondrial functions, such as mitochondrial biogenesis, thermogenesis, and fatty acid metabolism (2). Interestingly, mice overexpressing human GDF15 display increased life span (3). However, the role of GDF15 in promoting health or disease is still debated. There are in fact several evidences indicating that GDF15 levels are associated to progression of many diseases, such as cardiovascular diseases, insulin resistance and type 2 diabetes, neurodegeneration, renal chronic disease and cancer, but also to the limitation of the damage caused by stress and injuries (4–8). Accordingly, GDF15 has recently emerged as a potential biomarker for the aging process and many age-related diseases (2, 9–11). As far as muscle atrophy and sarcopenia, there is debate on whether GDF15 is to be considered protective or detrimental. Recent data from animal models showed that GDF15 is able to induce muscle fiber apoptosis (12, 13), but also the ablation of GDF15 resulted in an amplified skeletal muscle post exercise stress response, with a bigger increase of markers of muscle stress (Atf3, Atf6, and Xbp1s) (14). In humans, circulating GDF15 levels are significantly higher in subjects with sarcopenia or muscle atrophy (15–17) with respect to healthy subjects of comparable age. Recent studies demonstrate that GDF15 levels are negatively correlated with skeletal muscle mass index, hand-grip strength, muscle cross-sectional area and thickness (15, 18). Moreover, the loss of muscle mass observed in cachectic patients is mediated at least in part by the activity of GDF15 (19). On the other hand, it is known that physical exercise can effectively combat muscle atrophy, but is characterized by an increase in the level of circulating GDF15 (20–22). This could be explained by the fact that, as recently proposed, skeletal muscle is not the primary source of GDF15 (22). Finally, it is known that GDF15 is a stress molecule that is produced in response to mitochondrial, metabolic and inflammatory stresses (2, 23). To this regard, it is worth noting that a chronic state of low-grade inflammation, termed inflammaging, characterizes old people and is at the basis of many age-related diseases (24–26).

The aim of this study is to analyse the levels of circulating GDF15 in people of different age characterized by different levels of physical activity and to seek for correlation with hematological parameters related to inflammation. To this purpose, we studied three groups of subjects: 1. patients with chronic lower limb mobility impairment as a model of physical inactivity; 2. a group of amateur endurance cyclists as a model of physical activity; 3. and, age-matched subjects recruited from the general population, not actively exercising. Cyclists were sampled before and after a strenuous physical bout (a 130-km long distance road cycling race with a total uphill gradient of 1,871 m).

## Methods

### Subjects

In the present study, a total of 228 subjects in the age range from 18 to 83 years were recruited and divided into three different groups based on the level of physical activity: patients with lower limb mobility impairment (hereinafter patients), cyclists and controls. Patients suffered of coxarthrosis or hip dysplasia, causing them to be chronically unable to walk or exercising, and were therefore considered a model for prolonged physical inactivity. All subjects were further divided according to their age into the following groups: young, adult, late adult, old (Table 1). All subjects were enrolled in Italy in the framework of the following projects: the EU project MYOAGE for patients and controls, the “Novecolli Life” project promoted by Italian National Transplant Center for cyclists. The study protocols were approved by the Ethical Committee of Istituto Ortopedico Rizzoli, Bologna, Italy (ethical clearance no. 10823 issued on April 26, 2010) and by the Ethical Committees of the Italian Institute of Health (ethical clearance prot.no. 14/420 issued on March 7, 2014), respectively. All subjects signed an informed consent before entering the study. Age (>18 years) and ability to provide informed consent were inclusion criteria. Exclusion criteria were the presence of chronic kidney or liver diseases, unstable cardiovascular pathology, bleeding disorders, diabetes, neuromuscular disorders, systemic infections, major psychological problems, malignant neoplasia and/or a current therapy with immune suppressor drugs (like cyclosporine, methotrexate, glucocorticoids, etc.) or anticoagulant drugs, history of alcohol or drug abuse.

**Table 1:** Experimental sample description.

	**Cyclists**	**Controls**	**Patients**
***n*****°** **Young** Age range (average, ±SD)	**10** (2F, 8M) 18–39 yrs (30.3, ± 6.0)	**15** (9F, 6M) 18–39 yrs (29.1, ± 7.6)	**15** (5F, 10M) 24–39 yrs (33.0, ± 4.8)
***n*****°** **Adult** Age range (average, ±SD)	**32** (4F, 28M) 40–60 yrs (49.8, ± 5.8)	**21** (5F, 16M) 41–60 yrs (51.7, ± 6.5)	**20** (8F, 12M) 43–60 yrs (51.4, ± 5.6)
***n*****°** **Late adult** Age range (average, ±SD)	**5** (0F, 5M) 61–71 yrs (64.6, ± 6.0)	**32** (14F, 18M) 63–71 yrs (67.7, ± 2.8)	**17** (12F, 5M) 61–70 yrs (65.3, ± 2.5)
***n*****°** **Old** Age range (average, ±SD)	**____**	**43** (13F, 30M) 72–82 yrs (75.6, ± 3.1)	**12** (7F, 5M) 73–83 yrs (79.6, ± 3.5)

*Subjects are divided in four groups (in bold) according their age: Young, Adult, Late adult, Old. Each age group includes subjects with different levels of physical activity: Cyclists, Controls, Patients. The number (n°) of the subjects for each groups are reported in bold*.

### The Race

Briefly, the race, known as “Nove Colli,” is a long-distance cycling road race that takes place in Romagna (Forlì-Cesena and Rimini, Italy). The characteristics of the route were: length, 130 km; total uphill gradient, 1,871 m; uphill riding, 50 km over 4 hills; downhill riding, 46 km; flat terrain, 34 km; maximum riding time allowed, 7.5 h. For further details see Mosconi et al. (27, 28).

### Sampling and Data Collection

For patients and controls, blood was drawn in the morning after overnight fasting. All samples were processed immediately to collect plasma. For cyclists, the collection of venous blood (30 mL) samples was done at three different times: time 1 (T1), the day before the race, time 2 (T2), immediately after crossing the finish line, and time 3 (T3), 18–24 h after competing.

Plasma was obtained within 4 h from venipuncture by centrifugation at 2,000 g for 20 min at 4°C, rapidly frozen and stored at −80°C.

Blood cells and creatinine were measured by standard biochemical assays. White blood cell counts for cyclists (both T1 and T2) and patients divided for age group are presented in Supplementary Table 1. For cyclists, both at T1 and T2, three markers of cellular inflammation were calculated (Supplementary Table 2) as described below. The neutrophil-lymphocyte ratio (NLR) was calculated on the basis of absolute neutrophil (N; × 10^3^/*microL*) and lymphocyte (L; × 10^3^/*microL*) blood counts, using the formula: NLR = N/L. The platelet-lymphocyte ratio (PLR) was calculated on the basis of peripheral platelet (P; × 10^3^/*microLiter*) and lymphocyte (L; × 10^3^/*microL*) blood counts, using the formula: PLR = P/L. The systemic immune-inflammation index (SII) was calculated on the basis of peripheral platelet (P), neutrophil (N), and lymphocyte (L) blood counts, using the following formula: SII = P ^*^ N/L. All the inflammatory markers are ratios thus do not have a unit (29, 30).

Estimated glomerular filtration rate (eGFR) was calculated according to CKD-EPI (Chronic Kidney Disease Epidemiology Collaboration) equation based on serum creatinine, age, sex and ethnicity. (31). Body mass index (BMI) was calculated as weight in kilograms divided by the square of the height in meters (kg/m2). For patients, maximal quadriceps torque and *vastus lateralis* thickness were measured by using a Handifor® dynamometer and portable ultrasound (Mylab25, Esaote), respectively, as reported in (32).

GDF15 concentration was determined in plasma samples by ELISA assay using commercial kits, R&D (DGD150), according to the manufacturer's instructions. All the samples were measured in duplicate and the analyses were performed in a blind setup.

### Statistical Analysis

The data were analyzed with non-parametric tests since they did not follow a normal distribution. In particular, the comparisons among cyclists, controls and patients in the different age groups (young, adult, late adult) were performed by using Kruskal-Wallis test with a Steel *post-hoc* test, while the comparison between patients and controls in old group was performed by Mann-Withney test. To compare the GDF15 levels in cyclists at different times of the race (T1, T2, T3) we performed a Friedman test. The relationship among GDF15 levels and age, white blood cells, hematological markers of cellular inflammation (NLR, PLR, SII), creatinine, eGFR, and quadriceps torque were calculated by Spearman rank correlation test and regression analysis. Significance was accepted as p < 0.05. Data are expressed as mean ± SE. All data were analyzed using the SPSS 25.0 for Windows software (SPSS Inc.; Chicago, IL, USA).

## Results

### Plasma Levels of GDF15 Increase With Age and Are Negatively Associated With Active Lifestyle

**Figure 1:**
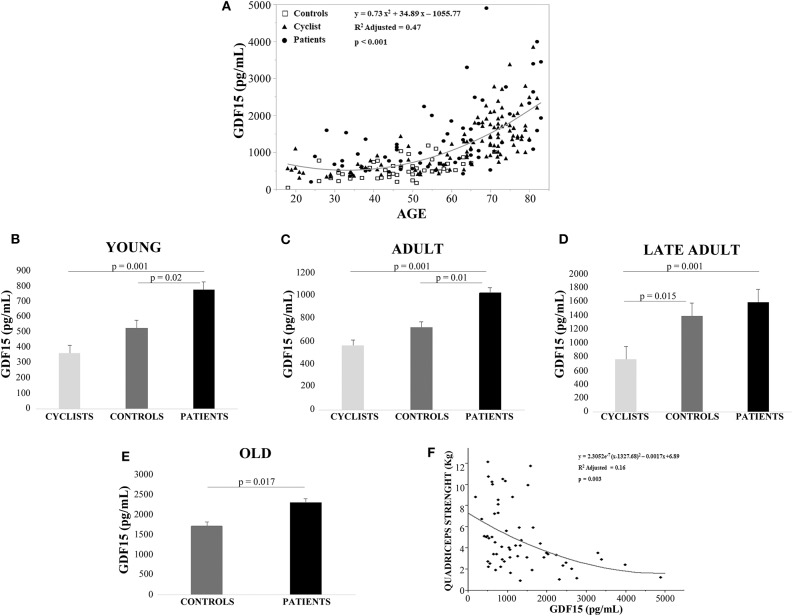
Plasma levels of GDF15 increase with age and are negatively associated with active lifestyle. **(A)** Regression analysis of circulating GDF15 and age in cyclists, controls and patients. **(B–D)** Circulating plasma levels of GDF15 in young cyclists (T1), controls and patients **(B)**; in adult cyclists (T1), controls and patients **(C)**; in late adult cyclists (T1), controls and patients **(D)**. Data are expressed as mean ± SE and *p*-values refer to Kruskal-Wallis test. **(E)** Circulating plasma levels of GDF15 in old controls and patients, data are expressed as mean ± SE and *p*-values refer to Mann-Whitney test. **(F)** Regression analysis of circulating GDF15 vs. quadriceps maximal torque normalized for age and *vastus lateralis* muscle thickness in patients.

**Figure 2:**
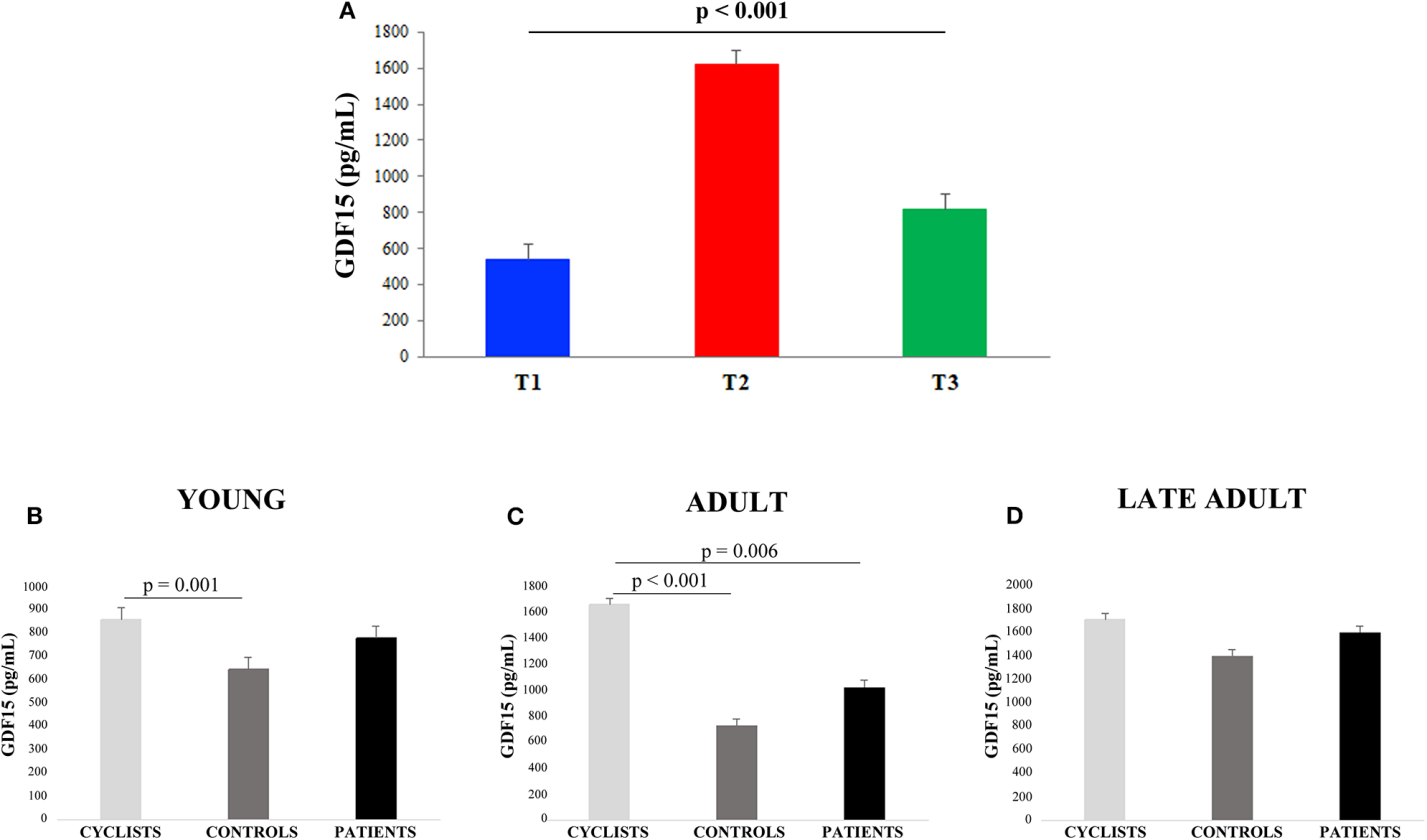
GDF15 plasma levels in cyclists before and after the race. **(A)** The differences among plasma GDF15 levels before the race (T1), immediately after the race (T2), and after 18–24 h from the race (T3) were analyzed. Data are expressed as mean ± SE *p*-values refer to Friedman test. **(B–D)** Circulating plasma levels of GDF15 in young cyclists (T2), controls and patients **(B)**; in adult cyclists (T2), controls and patients **(C)**; in late adult cyclists (T2), controls and patients **(D)**. Data are expressed as mean ± SE and *p*-values refer to Kruskal-Wallis test.

Linear regression analysis showed that GDF15 plasma levels were significantly associated with age for all the 228 subjects (Figure 1A). Spearman rank correlation coefficient and *p*-value are: ρ= 0.741, *p* < 0.0001. This age-related increase of plasma GDF15 was evident irrespective to the level of physical activity of the subjects, confirming previous data on GDF15 and age (10). However, when the subjects were subdivided on the basis of their age and level of physical activity (as described in Materials and Methods Section), the plasma levels of GDF15 were significantly higher in inactive patients and significantly lower in active people such as cyclists with respect to control subjects, in young, adult and late adult people (Figures 1B–D). For ages over 72 years, only patients and controls were available but also in this case the same trend was observed (Figure 1E). The relationship between physical activity and GDF15 was also analyzed considering BMI as a covariate, and the results remained the same (data not shown). Therefore, it seems that the level of physical activity determines the plasma levels of GDF15 at any age. For patients, the values of maximal quadriceps torque normalized on *vastus lateralis* muscle thickness were available, and these values, considering age as a covariate, resulted inversely correlated to GDF15 plasma levels; Spearman rank correlation coefficient and *p*-value are: ρ = −0.449 and *p* < 0.0001 (Figure 1F). Similar results were obtained when quadriceps torque was normalized on total BMI (data not shown). However, as already reported, a strenuous physical exercise (in our case, the long-distance cycling race) is able to cause a dramatic increase of plasma GDF15, as evidenced by the difference between T2 (immediately after the race) and T1 (before the race) (Figure 2A). After 18–24 h from the race (T3), the levels of GDF15 tend to return at baseline level, even if these levels remain significantly higher than T1 (Figure 2A). We also compared the levels of GDF15 at T2 with those observed in controls and patients of similar age. In the group of young subjects GDF15 levels resulted significantly higher with respect to controls and similar to patients (Figure 2B), while in the adult group the GDF15 levels resulted significantly higher with respect to controls and patients (Figure 2C). In the group of late adults, no difference was present (Figure 2D).

### Relationship Among Plasma Levels of GDF15 With Hematological Parameters Related to the Inflammatory Response and Renal Function

**Figure 3:**
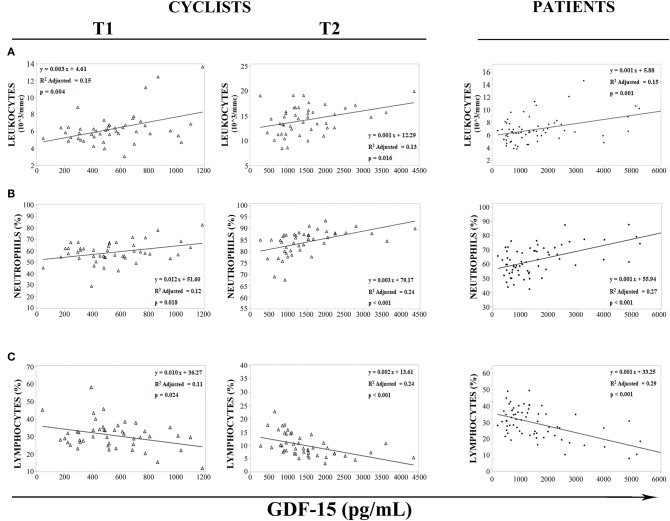
Regression analysis of GDF15 plasma levels with hematological parameters. **(A–C)** Regression analysis of circulating GDF15 levels and leukocytes **(A)**, neutrophils **(B)**, lymphocytes **(C)**, in cyclists before the race (T1), immediately after the race (T2), and in patients.

It is known that GDF15 is responsive to mitochondrial stress and inflammation, two conditions that apply under strenuous physical exercise (33). We then sought for associations with hematological parameters associated to an inflammatory response. In cyclists, before (T1) and immediately after (T2) the race, regression analysis has shown an association between white blood cells and GDF15 plasma levels (Figures 3A–C). Moreover, GDF15 plasma levels significantly and positively correlated with the number of total leukocytes (T1: ρ = 0.311 and *p* = 0.036, T2: ρ = 0.343 and *p* = 0.019) (Figure 3A) and neutrophils (T1: ρ = 0.346 and *p* = 0.018, T2: ρ = 0.621 and *p* < 0.0001) (Figure 3B), and negatively with lymphocytes (T1: ρ = −0.332 and *p* = 0.024, T2: ρ = −0.613) (Figure 3C). Interestingly, the same associations were found for patients (Figures 3A–C) and in this case the Spearman rank correlation coefficients were similar to those observed for cyclists' T2 (leukocytes: ρ = 0.388 and *p* = 0.001; neutrophils: ρ = 0.519 and *p* < 0.0001; lymphocytes: ρ = −0.536 and *p* < 0.0001). Therefore, it seems that a strenuous physical exercise produces a transient state of stress that, as far as GDF15 and inflammatory parameters, is similar to that present in patients.

**Figure 4:**
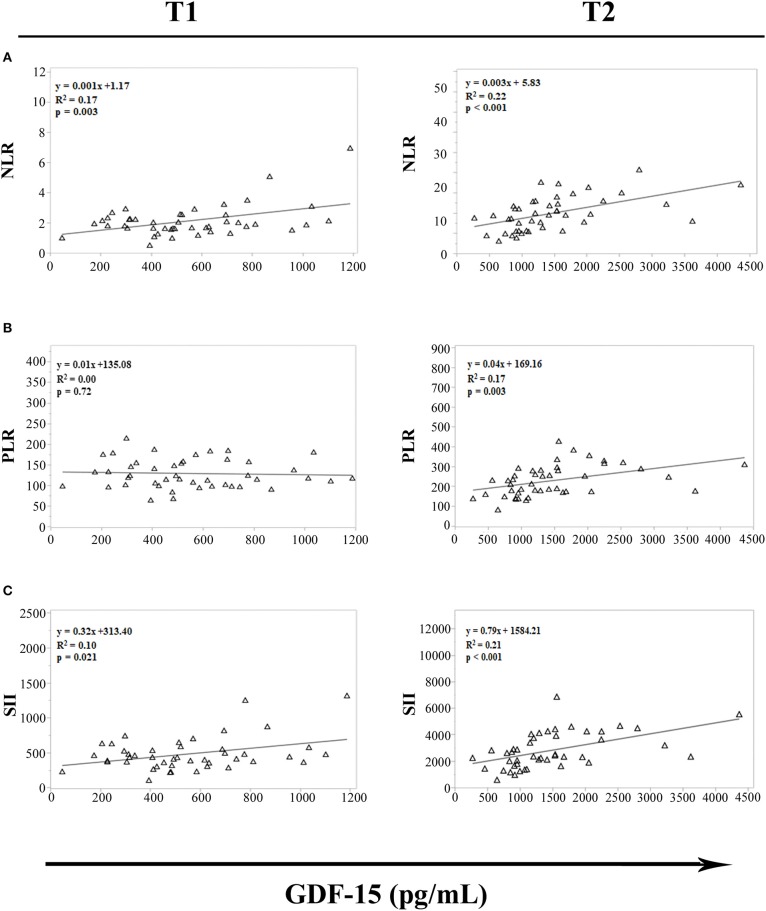
Regression analysis of GDF15 plasma levels with hematological markers of cellular inflammation in cyclists before (T1) and immediately after (T2) the race. **(A)** Regression analysis of circulating GDF15 levels and neutrophil/lymphocyte ratio (NLR); **(B)** regression analysis of circulating GDF15 levels and platelet/lymphocyte ratio (PLR); **(C)** regression analysis of circulating GDF15 levels and systemic immune-inflammation index (SII).

Recently, several studies have proposed NLR, PLR and SII as white blood-cell-based inflammatory markers. The levels of these inflammatory indices are in fact elevated in individuals with chronic inflammation and several age-related pathologies (29, 30). To evaluate whether strenuous exercise influences the relationship between GDF15 plasma levels and these inflammatory markers, a regression analysis in cyclists at T1 and T2 was performed. We observed significant association between GDF15 and NLR, and GDF15 and SII, both at T1 and T2 (Figures 4A,C); while the association between GDF15 and PLR is present only at T2 (Figure 4B). Interestingly, these associations are stronger at T2, as also confirmed by Spearman's correlation analysis. In this case in fact, only at T2 the levels of GDF15 significantly correlate with these markers (NLR: ρ = 0.616 and *p* < 0.0001; PLR: ρ = 0.567 and *p* < 0.0001; SII: ρ = 0.580 and *p* < 0.0001).

**Figure 5:**
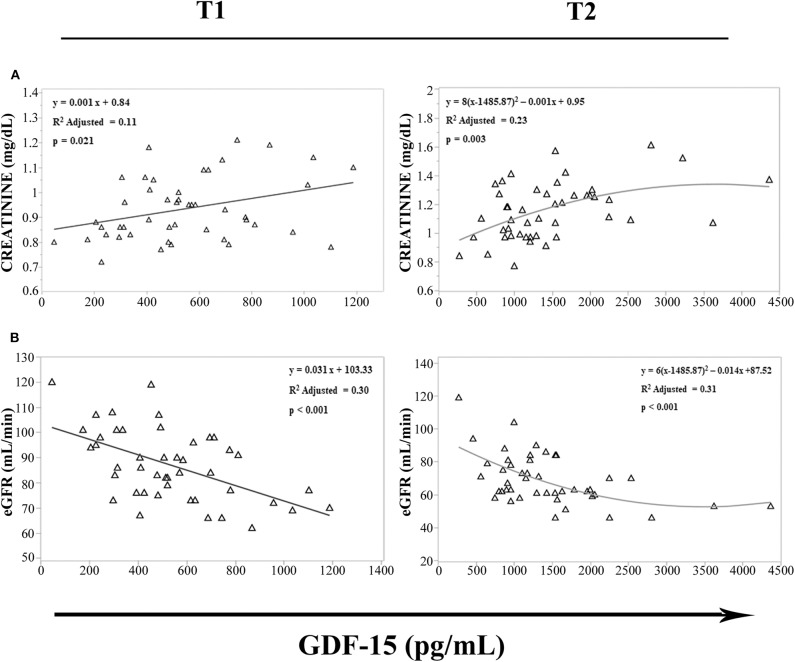
Regression analysis of GDF15 plasma levels with estimated glomerular filtration rate (eGFR) and creatinine. **(A,B)** Regression analysis of circulating GDF15 and eGFR **(A)**, creatinine **(B)** in cyclists before the race (T1), immediately after the race (T2).

It has been recently reported that upon metformin treatment, GDF15 is increased in distal intestine and kidney (34), and an intense sport exercise induces acute renal stress (35), with an increase in creatinine levels and a decline of estimated glomerular filtration rate (eGFR), as indices of renal function (36). We then evaluated in cyclists at T1 and T2 the possible relationship among GDF15 levels, creatinine, and eGFR. We observed that GDF15 levels were associated with creatinine (Figure 5A) and eGFR (Figure 5B). Furthermore, according to the Spearman's correlation analysis, GDF15 plasma levels, both at T1 and T2, positively correlated with creatinine (T1: ρ = 0.316 and *p* = 0.032, T2: ρ = 0.422 and *p* = 0.003), and negatively with eGFR (T1: ρ = −0.506 and *p* < 0.0001, T2: ρ = −0.486 and *p* = 0.001). These results suggest that the elevation of GDF15 observed during a strenuous physical exercise is associated with renal stress.

## Discussion

GDF15 is a cytokine that was considered expressed only by a limited number of tissues, such as liver, lung and kidney, as well as by the placental trophoblast (37). More recently it has been demonstrated that GDF15 is responsive to mitochondrial stresses (38), and, according to the theory that aging is associated with increased mitochondrial dysfunction, elevated circulating levels of GDF15 are found in elderly people and centenarians (9–11). As it is known that strenuous exercise induces GDF15 expression (20–22), it has been hypothesized that also skeletal muscle can be a source of this cytokine. Accordingly, GDF15 is expressed in muscles from mouse models of aging and inactivity (12, 14). However, this idea has been recently challenged, as the concentrations of GDF15 during a physical exercise resulted similar in arterial and venous blood across the exercised leg (22). Our results are in favor of the idea that skeletal muscle is not the primary source of GDF15, as its basal levels are lower in actively exercising people like cyclists with respect to age-matched controls. However, this does not exclude at all that skeletal muscles can produce a little amount of GDF15. Moreover, after a bout of strenuous physical activity, the levels of GDF15 are correlated with markers of kidney injury, suggesting that, according to literature data (34), the elevation of GDF15 may be a response to an injury to other organs, including the kidney.

Whether GDF15 is beneficial or detrimental for skeletal muscle is still debated. It has been previously reported that GDF15 causes anorexia/cachexia via its impact on energy metabolism (18) and, accordingly, it is found inversely associated with muscle mass (39). Moreover, even though its receptor, GDNF family receptor α-like (GFRAL), has not been found expressed in muscle (40) GDF15 is able to induce muscle fiber apoptosis via phosphorylation of STAT3 (13). On the other side, as mentioned, the GDF15 knockout determines the elevation of markers of muscle stress (Atf3, Atf6, and Xbp1s) upon exercise (14). Our data suggest that GDF15 is inversely associated with muscle health, as it is elevated in patients with lower limb mobility impairment and inversely associated with their quadriceps maximal torque. It is at present unclear whether this association is causal or not. In order to reconcile our data with those demonstrating a beneficial role for GDF15, we can hypothesize that transient peaks of GDF15 are stimulatory/homeostatic, whereas long-lasting elevated systemic levels can turn detrimental. This could be the case of patients with lower limb mobility impairment.

Another alternative possibility is that GDF15 acts in synergy or in opposition with other factors. Interestingly, some samples from cyclists used in this study were previously assayed for the expression of inflammatory mediators such as IL-6, TNF-α, and IFN-γ that resulted dramatically increased after the race (33). To this regard, it is worth noting that GDF15 is also responsive to inflammation, mostly *via* p53 (41). It has indeed been shown that GDF15 is a direct target gene of p53. Recently, it has been demonstrated that GDF15 is necessary for tolerance to inflammation induced by viral or bacterial infections (23). It has also a clear anti-inflammatory activity in experimental models of liver injury and myocardial infarction (42, 43). In particular, GDF15 attenuates the LPS-induced production of classical pro-inflammatory cytokines such as TNF-a, IL-1β and IL-6 in Kupffer cells (42), and is able to inhibit the chemokine-activated leukocyte arrest on the myocardial endothelium of infarcted heart (43). Here we show that GDF15 levels are clearly associated with hematological parameters related to inflammation, *i.e*., increased number of leukocytes (in particular neutrophils) and decreased number of lymphocytes, in both cyclists and patients. Thus, it is possible that the net effect of GDF15 on muscle health depends on the fine interaction with inflammatory mediators.

Given its responsiveness to inflammation and reported anti-inflammatory effects, the elevated levels of GDF15 can be interpreted as an automatic mechanism to blunt the detrimental effects of inflammation (acute like a strenuous bout of physical activity, or chronic like that present in inactive patients). It has been previously reported that a chronic, subclinical inflammation (inflammaging) is a typical feature of old people. Therefore, it is tempting to speculate that GDF15 is elevated in the elderly at least in part as a consequence of inflammaging, and that GDF15 could be added to the list of anti-inflammaging mediators. Elderly people are also characterized by a loss of muscle mass and power (44), and we have reported that GDF15 levels are very high in old people and centenarians (10). Therefore, due to its wasting activity on muscle, it is thus conceivable that the elevated levels of GDF15 in elderly people and particularly in the oldest old can be a *trait-d'union* between inflammaging and the observed loss of muscle mass and power.

Finally, it can be hypothesized that people with less inflammaging have consequently a lower production of GDF15. A corollary of this hypothesis is that the positive effects of GDF15 are likely not enough to overcome the detrimental ones brought by inflammaging. These considerations possibly indicate GDF15 as a target for future pharmacological or life-style interventions to implement healthy aging and longevity, whose goal would be to obtain the beneficial effects of GDF15 avoiding the detrimental ones. Future studies are needed in this perspective.

## Data Availability Statement

The datasets generated for this study are available on request to the corresponding author.

## Ethics Statement

The study protocols were approved by the Ethical Committee of Istituto Ortopedico Rizzoli, Bologna, Italy (ethical clearance no. 10823 issued on April 26, 2010) and by the Ethical Committees of the Italian Institute of Health (ethical clearance prot.no. 14/420 issued on March 7, 2014), respectively. The patients/participants provided their written informed consent to participate in this study.

## Author Contributions

MCo: patients' enrollment, data generation and collection, statistical analysis, and writing of the manuscript. MM: analysis of GDF15 and manuscript revision. GM: cyclists' enrollment and manuscript revision. AC: analysis of GDF15. MCa and VT: clinical data collection of cyclists. AS: manuscript revision. CF: critical discussion. SS: analysis of the data and writing of the manuscript. All authors approved the final version of the manuscript.

## Conflict of Interest

The authors declare that the research was conducted in the absence of any commercial or financial relationships that could be construed as a potential conflict of interest.
