# Supplementary material for: GDF15 Plasma Level Is Inversely Associated With Level of Physical Activity and Correlates With Markers of Inflammation and Muscle Weakness
Source: Front Immunol. 2020 May 12;11:915. doi: 10.3389/fimmu.2020.00915 (PMC7235447; doi:10.3389/fimmu.2020.00915)
Supplement: Supplementary file 1 [file Table_1.DOCX]

|  | **CYCLISTS**  T1 T2 | | **PATIENTS** |
| --- | --- | --- | --- |
| **White blood cells**  (MEAN ± SD) | **YOUNG** | | |
| Leukocytes (10^3/mmc) | 6.48 ± 1.87 | 13.35 ± 3.25 | 6.92 ± 2.04 |
| Neutrophils (10^3/mmc)  (%) | 3.61 ± 1.68  54.02 ± 9.27 | 10.63 ± 3.40  78.33 ± 8.51 | 4.51 ± 1.76  63.57 ± 9.40 |
| Lymphocytes (10^3/mmc)  (%) | 2.11 ± 0.40  33.79 ± 7.52 | 1.79 ± 1.55  14.43 ± 6.95 | 1.78 ± 0.44  26.96 ± 7.44 |
| Monocytes (10^3/mmc)  (%) | 0.38 ± 0.14  5.90 ± 1.04 | 0.69 ± 0.17  5.24 ± 1.05 | 0.46 ± 0.11  6.99 ± 1.99 |
| Basophils (10^3/mmc)  (%) | 0.04 ± 0.02  0.62 ± 0.38 | 0.04 ± 0.03  0.33 ± 0.30 | 0.03 ± 0.02  0.47 ± 0.18 |
| Eosinophils (10^3/mmc)  (%) | 0.23 ± 0.21  3.90 ± 3.67 | 0.09 ± 0.16  0.89 ± 1.65 | 0.14 ± 0.08  2.01 ± 1.27 |
|  | **ADULT** | | |
| Leukocytes (10^3/mmc) | 6.07 ± 1.71 | 13.84 ± 3.00 | 6.44 ± 1.31 |
| Neutrophils (10^3/mmc)  (%) | 3.61 ± 1.55  58.51 ± 8.66 | 11.85 ± 2.97  84.99 ± 4.41 | 3.81 ± 1.07  59.45 ± 8.67 |
| Lymphocytes (10^3/mmc)  (%) | 1.82 ± 0.55  30.60 ± 7.83 | 1.18 ± 0.37  9.07 ± 3.70 | 1.89 ± 0.64  29.04 ± 8.25 |
| Monocytes (10^3/mmc)  (%) | 0.37 ± 0.10  6.16 ± 1.31 | 0.68 ± 0.21  4.91 ± 1.22 | 0.52 ± 0.18  8.04 ± 2.01 |
| Basophils (10^3/mmc)  (%) | 0.03 ± 0.01  0.43 ± 0.20 | 0.02 ± 0.01  0.18 ± 0.1 | 0.03 ± 0.01  0.44 ± 0.17 |
| Eosinophils (10^3/mmc)  (%) | 0.14 ± 0.09  2.36 ± 1.44 | 0.05 ± 0.04  0.36 ± 0.33 | 0.19 ± 0.14  2.99 ± 2.06 |
|  | **LATE ADULT** | | |
| Leukocytes (10^3/mmc) | 7.40 ± 2.93 | 14.63 ± 0.95 | 6.68 ± 2.36 |
| Neutrophils (10^3/mmc)  (%) | 4.94 ± 2.80  64.04 ± 11.54 | 12.66 ± 0.74  86.56 ± 0.98 | 4.07 ± 2.01  58.80 ± 8.36 |
| Lymphocytes (10^3/mmc)  (%) | 1.79 ± 0.30  26.30 ± 8.42 | 1.20 ± 0.16  8.2 ± 0.70 | 1.94 ± 0.59  30.79 ± 7.62 |
| Monocytes (10^3/mmc)  (%) | 0.36 ± 0.10  5.08 ± 1.27 | 0.63 ± 0.06  4.28 ± 0.23 | 0.47 ± 0.18  7.2 ± 1.76 |
| Basophils (10^3/mmc)  (%) | 0.03 ± 0.02  0.34 ± 0.17 | 0.02 ± 0.01  0.16 ± 0.05 | 0.05 ± 0.09  0.55 ± 0.23 |
| Eosinophils (10^3/mmc)  (%) | 0.18 ± 0.12  2.72 ± 2.23 | 0.03 ± 0.01  0.2 ± 0.07 | 0.30 ± 0.60  2.67 ± 1.40 |

**Supplementary table 1:** White blood cell counts in cyclists (before and after a strenuous physical bout, T1 and T2) and patients

**Supplementary table 2:** Hematological markers of cellular inflammation in cyclists; neutrophil/lymphocyte ratio(NLR); platelet/lymphocyte ratio (PLR); systemic immune-inflammation index (SII).

| Haematological  Markers | T1  (mean ± SD) | T2  (mean ± SD) | T3  (mean ± SD) |
| --- | --- | --- | --- |
| **NLR** | 2.1 ± 1.0 | 10.1 ± 5.0 | 2.3 ± 0.8 |
| **PLR** | 131.3 ± 38.7 | 237.8 ± 108.2 | 140.6 ± 32.6 |
| **SII** | 487.0 ± 245 | 2909.3 ± 1891.8 | 554.8 ± 191.7 |
| Age: range 18-71; mean ± SD: 47 ± 11 | | | |
